# A simple approach for the management of a ruptured endobronchial cuff of the double-lumen tube using an automatic cuff pressure controller: a case study

**DOI:** 10.1186/s40981-022-00551-2

**Published:** 2022-08-08

**Authors:** Akinobu Hibino, Tomohiro Yamamoto

**Affiliations:** Division of Anesthesiology, Niigata University Graduate School of Medical and Dental Sciences, 1-757, Asahimachi-dori, Chuo ward, Niigata, 951-8510 Japan

To the Editor,

Double-lumen endotracheal tube (DLT) cuff leakage hinders one-lung ventilation (OLV) and risks safety of the surgical procedure; therefore, this should be addressed immediately. The ideal management for such cases is DLT replacement [1]. However, DLT replacement may be complicated, especially when the patient is in the lateral position or the surgery has already begun. Several methods of handling endotracheal tube (ETT) cuff leakage have been reported, highlighting the principle of pumping air from the pilot balloon to the cuff continuously using an air-filled syringe attached to a syringe pump [2–4]. Despite this, the cuff pressure should always be measured and controlled to prevent barotrauma, which is an important complication in a syringe and syringe pump system [Groves, 2010 #292].

A 35-Fr left-sided DLT (COOPDECH Double-Lumen Endobronchial Tube; Daiken Medical Co., Ltd., Osaka, Japan) was used for OLV in a 76-year-old female for right lower pulmonary lobectomy via video-assisted thoracic surgery. After the patient was positioned in the left lateral position, we confirmed the position of the blue endobronchial cuff using a bronchoscope. However, upon inserting the endoscope into the right chest cavity after initiating surgery, the video monitor showed that the right lung was slightly ventilated. On further investigation, the pilot balloon of the blue endobronchial cuff revealed an air leakage, which we suspected was due to damage as it quickly deflated even after additional air was administered using a syringe. When we checked the status of the blue endobronchial cuff using the bronchoscope, we observed bubbles surrounding the area, confirming our suspicion of endobronchial cuff damage.

Although we initially considered replacing the DLT, it was difficult since the patient was in the left lateral position and the surgery had already begun. As an alternative solution, we decided to use the SmartCuff® (Murata Manufacturing Company, Ltd., Kyoto, Japan), a portable, cordless, battery-powered (145 g) device that automatically adjusts the cuff pressure at the set value by pumping air to the pilot balloon continuously.

After connecting the SmartCuff® to the blue pilot balloon of the DLT and turning it on, the set pressure was changed to 20 cm H_2_O using the settings option by pressing the plus and minus buttons accordingly. Following this, the SmartCuff® automatically maintained this cuff pressure at the set value (Fig. 1). This method enabled us to complete the 5-h surgery without any complications, as OLV was achieved immediately after the use of the SmartCuff®. After extubation, we checked the blue endobronchial cuff of the DLT and found a tear measuring approximately 1 mm (Fig. 2). The SmartCuff® is beneficial in these cases, as it has a large air supply capacity of up to 95 ml/min, which could also help in addressing larger cuff leakages to some extent. The SmartCuff® can set a wide range of cuff pressure settings from 5 to 80 cm H_2_O. The only disadvantage in using this device is that an alarm will go off every 10 s if the cuff pressure does not stabilize at the set value, which requires the user to silence the alarm frequently.

**Fig. 1:**
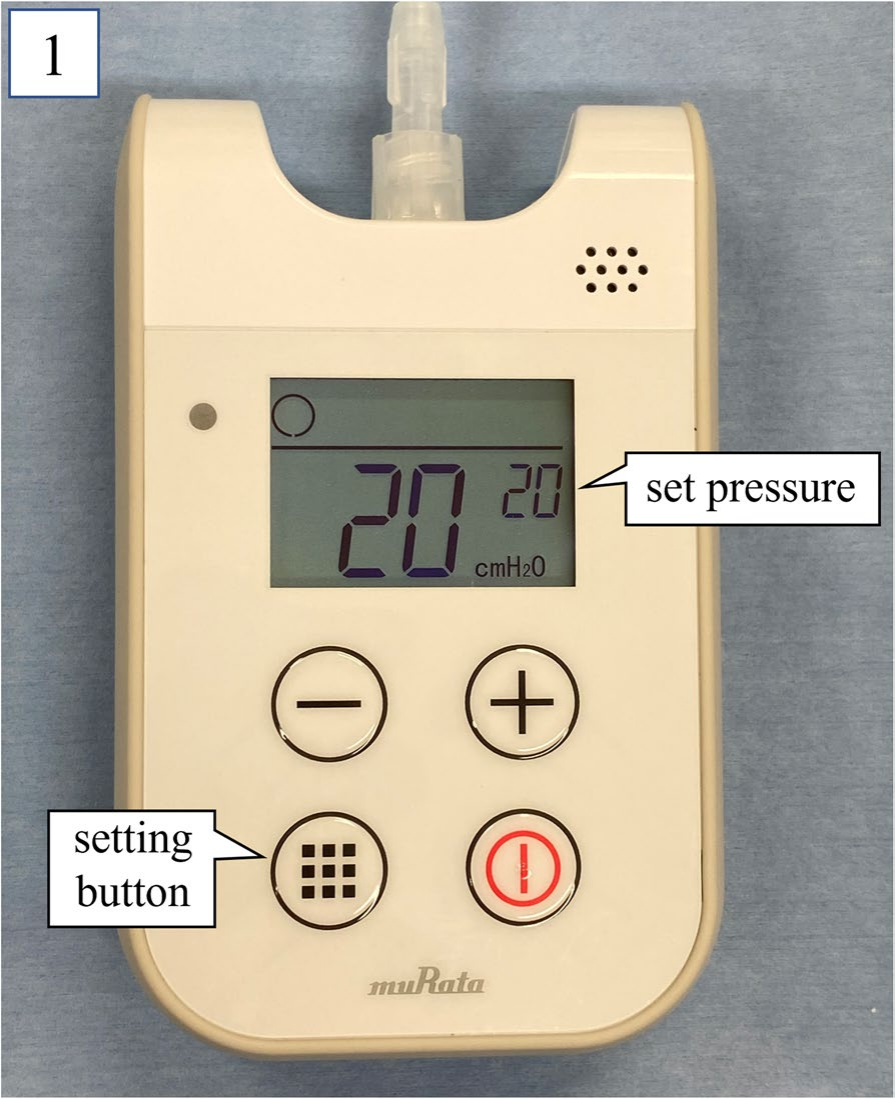
SmartCuff®, a portable cordless device that is powered by batteries weighing only 145 g. The SmartCuff® automatically adjusts the cuff pressure of the endotracheal tube, which in turn responds to changes in the cuff pressure by controlling air delivery to the pilot balloon, enabling cuff pressure maintenance at the set value

**Fig. 2:**
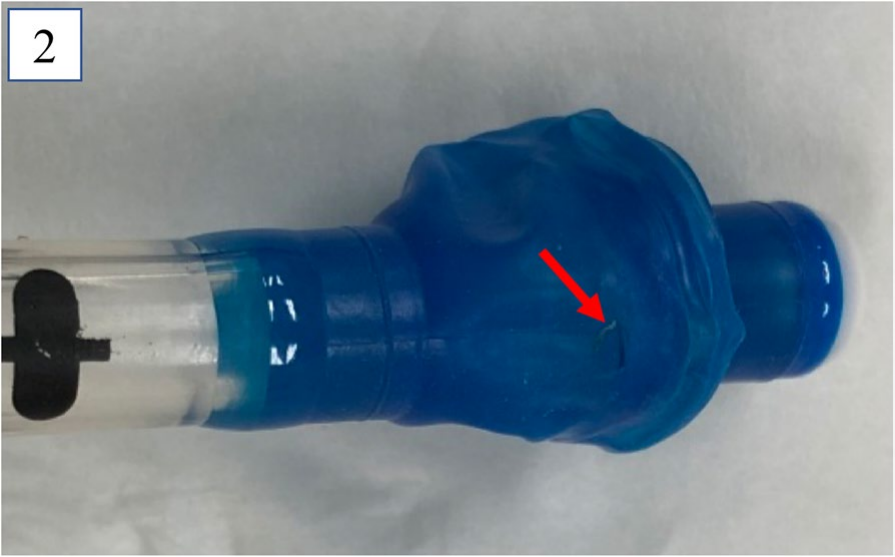
The damaged part of the blue endobronchial cuff of the double-lumen endotracheal tube. The blue endobronchial cuff of the double-lumen endotracheal tube shows a tear measuring approximately 1 mm

This technique in utilizing the SmartCuff® may be used as an alternative solution to manage complicated cases of ruptured cuff of an ETT, including DLTs. Since air is continuously injected into the bronchial cuff of the DLT and leaks to both the distal and proximal sides of the cuff, the leaked air may accumulate on the unventilated lung and consequently increase its internal pressure. Although the degree of this risk depends on the amount of air delivered, anesthesiologists must still be aware of this possibility.

## Data Availability

Not applicable.

## Abbreviations

DLT: Double-lumen endotracheal tube
OLV: One-lung ventilation
ETT: Endotracheal tube
